# Surgical fenestration and rehabilitation of a sports traumatic non-union ischial tuberosity fracture – Case report

**DOI:** 10.1016/j.ijscr.2018.11.018

**Published:** 2018-11-17

**Authors:** Jens Erik Jorgensen, Carsten M. Mølgaard, Jens Kristinsson

**Affiliations:** a Physiotherapy Clinic, Sofiendalsvej 92A, 9200, Aalborg SV, Denmark; b Department of Orthopaedic Surgery, Aalborg University Hospital, Hobrovej 18-22, 9000, Aalborg, Denmark; c Department of Physio and Occupational Therapy, Aalborg University Hospital, Hobrovej 18-22, 9000, Aalborg, Denmark

**Keywords:** Non-union, Sports injury, Trauma, Fenestration, Adolescent apophyseal injury, Ischial tuberosity avulsion

## Abstract

**Introduction:**

Ischial tuberosity fractures and complications may be an under recognized diagnosis in adolescent athletes. Operative interventions differ and may include anchor re-fixations, resections and osteostimulating drilling and partial hamstring releases. This case report illustrates a novel and less invasive management of a non-union following a proximal ischial tuberosity avulsion. This approach has to our knowledge not previously been described.

**Presentation of case:**

The patient, a 14 years old female athlete, was complaining of sharp pain in the right side of the groin region, after an acute injury during a handball game. The pain was concentrated at the insertion of the adductor muscle group to the superior pubic ramus and the pubic symphysis. She was referred to our orthopaedic sports clinic after 6 months of unsuccessful conservative treatment. An MRI scan showed an ischial tuberosity non-union with a displacement less than 10 mm. Nine months after injury a surgical procedure was performed with the patient under general anaesthesia. An ultrasound guided fenestration of the non-union of the ischial tuberosity with a 1.6 mm Kirshner wire was performed. The enthesis was fenestrated 10 times, using a 1.2 mm syringe. Finally, a 5 ml local anaesthetic was injected in the area. 17 months after the trauma and 8 months postoperatively the patient had a full return to normal day activities including pain free squats and lunges. Radiologically the non-union was healed.

**Conclusion:**

This novel and less invasive surgical procedure may therefore be seen as a possible treatment option to non-union of the ischial tuberosity with minimal displacement.

## Highlights

• Avulsion fracture is always a differential diagnosis in proximal hamstring pain in adolescence.
• In acute nondistinctive cases of the pelvis CT, MRI, or even ultrasound may help to make the correct diagnosis.
• Consider fenestration of avulsion fractures if no or small progression with conservative treatment after 3–4 months after injury.

## 1 Background

This case report illustrates the management of a non-union following a proximal ischial tuberosity avulsion in a young female team handball goalkeeper that to our knowledge has not been described in the literature. The case has been reported in line with the SCARE criteria [1]

Diagnosing and management of proximal hamstring injuries in adolescent athletes can be challenging for the sports medicine practitioner [[2], [3], [4]]. Avulsion injuries affect the secondary centres of ossification primarily in children and adolescents between the ages of 11–17 years [5]. When ischial tuberosity avulsions are encountered the hamstrings are often the cause of the avulsion fractures, due to the stress produced by the pulling force to the bone [6]. Injury to the pelvic apophysits occurs more often in boys than in girls with a ratio of 2:1; the greatest injury incidence approximately at the age 13 years for boys and 12 years for girls [2,5]. Lower extremity avulsion fractures are uncommon in the paediatric population and can be misdiagnosed without proper imaging and/or clinical suspicion for these injuries. Less often, avulsion fractures occur in the tibial tubercle, calcaneus, and greater and lesser trochanters [3]. Imaging is an important issue in diagnosing these patients. Plain radiographs can be interpreted as negative in children, when an apophyseal avulsion is non-displaced or when the apophysis is unossified. In such cases, ultrasound, CT and MRI may prove helpful [6]. In patients with ambiguous radiographic findings and continued pain despite conservative treatment, MR imaging is the current imaging modality of choice [3]. Routine use of CT should be limited to the evaluation of complex fractures (comminuted fracture, higher grade physeal separation, and intra-articular fracture fragments) and for surgical planning [4].

The patient was a 14-year-old multi-sport female athlete (soccer and handball) complaining of sharp pain in the right side of the groin region, after an acute injury during a handball game. The injury caused by a sudden forceful eccentric contraction of the hamstrings [7], in a split movement, attempting to save a shot at goal. The pain was concentrated at the insertion of the hamstring and adductor magnus muscle, at the ischial tuberosity. Aggravating activities included walking, stair climbing, and prolonged sitting. Past medical history was unremarkable. Her general practitioner (GP) had suggested resting the injury. Due to ongoing pain and lack of functional progression, she consulted a physiotherapist in private practice, two weeks after the initial trauma. At the initial examination, her symptoms were beginning to subside, and daily function was improved, therefore conservative treatment was continued [7], focussing on strengthening the hip rotators, flexors, and adductors. The conclusion of the physiotherapy examination was a possible trauma to the pubic symphysis, and subsequently referred to a sports physician for further evaluation, and conservative treatment was prescribed.

Almost three months after the initial trauma, she felt a sharp pain at the initial injury site, and her functional capability decreased significantly. After a renewed specialist consultation, she was referred to MRI. The MRI revealed an avulsion fracture of the tuber ischiadicum, and consequently she was referred on to the orthopaedic sports clinic for further evaluation.

**Fig. 1:**
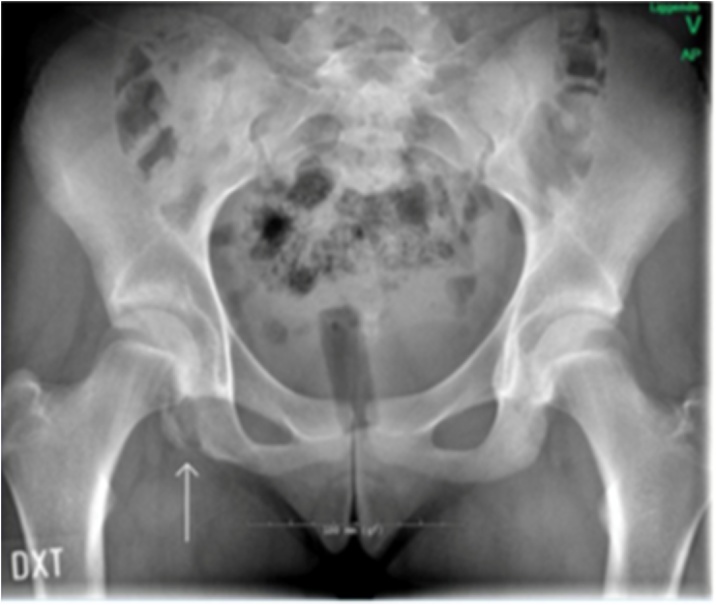
X-ray taken 6 months after symptom debut. Arrow show fracture line at the right ischial tubercle. Displacement was less than 10 mm.

Initial examination at the sports clinic revealed that she was unable to do a 20 cm step up, run, cycle, nor walk 1.5 km. Full knee and range of motion (ROM). Distinct palpation pain at the medial side of the right Ischial tubercle. Maximum internal and external hip rotation produced sharp pain at the Ischial tubercle. Isometric knee flexion and extension strength, tested by handheld dynamometer at 60 ° of knee flexion, exhibiting a 50% decrease in strength, compared to the left leg. The patient's Lower Extremity Functional Scale (LEFS) score was 38/80, with a score of 80 translating to no deficits. X-ray imaging was prescribed, which confirmed the fracture seen on MRI. (Fig. 1)

There remains variability in the definitions of fracture-healing [8]. In the study conducted by Bhandari and colleagues [8], surgeons felt confident of predicting non-union fractures of the pelvic rami by the fourteenth week. Pain on weight-bearing (more than three months after fracture for a delayed union and more than six months after fracture for a non-union) was endorsed as the most consistent predictor of delayed union and non-union fractures [8].There is as yet no consensus of the definition of non-union fractures [[8], [9], [10], [11]],as several factors need to be considered in defining delayed or non-union fractures, rather than a specific timeline [8]. This avulsion fracture may therefore be classified as a non-union.

An ultrasound-guided steroid injection (1 mL Depomedrol,3 mL Lidocaine) was administered at the insertion of the medial hamstring and adductors to the ischial tuberosity. After ten minutes, the patient could do a step-up and a full squat with no pain, however the effect subsided after two weeks. The rationale for applying the steroid injection being to eliminate the possibility of referred pain.

**Fig. 2:**
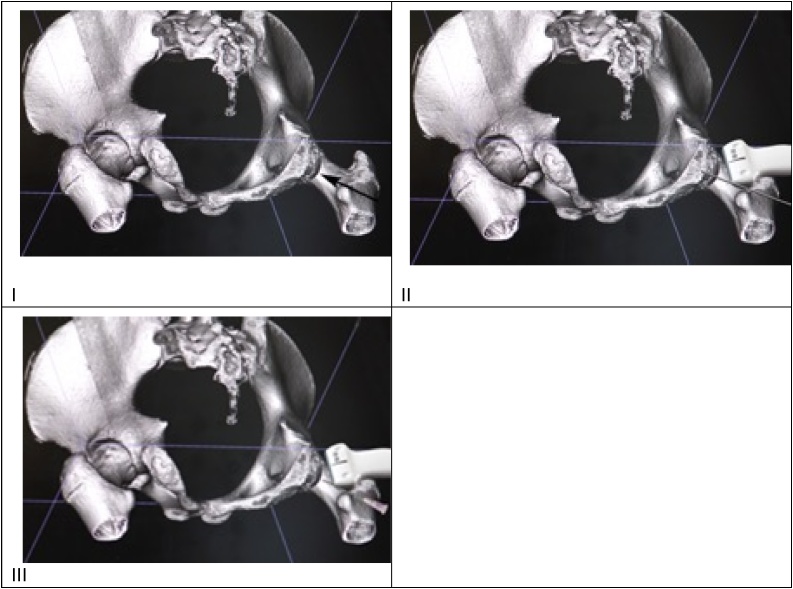
Schematic presentation of the surgical intervention. I. 3D scan. Arrow show fracture. II. Fenestration with K-wire. III. Fenestration at enthesis.

Nine months after injury a surgical procedure was performed with the patient in general anaesthesia and lying on her left side to induce a strong healing response at the non-union. An ultrasound guided percutaneous fenestration with K-wire 1.6 mm was repeated 6 times at the avulsion fracture. The enthesis was fenestrated 10 times, using a 1.2 mm syringe. Finally, a 5 ml local anaesthetic was injected in the area. The procedure lasted 20 min in total. The novelty of the method being the use of a K-wire in repairing a non-union, as opposed to the percutaneous needle tenotomy [12,13], in which percutaneous needle fenestration is performed at the fracture site, inducing a healing response prior to considering operative fixation of the non-union fracture [13]. Other methods of operative interventions may include reconstructive anchor re-fixations, resection procedures of the non-united fragments, resections and osteostimulating donor site drilling and partial hamstring releases for traumatic hamstring syndrome [14] (Fig. 2).

**Fig. 3:**
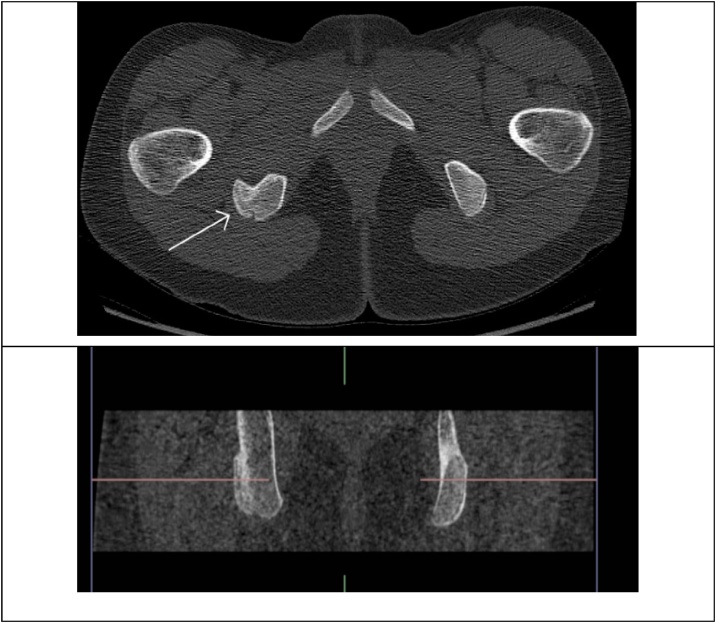
Arrow show healing of fracture line at the right ischial tubercle.

Rehabilitation in the primary sector was adapted by the work of Shoensee and Nilsson [5]. Following the fenestration with K-wire, the patient was placed on crutches and was instructed to remain non-weight bearing and avoid sitting directly on her right buttock for two weeks. The rehabilitation progression was based on proposed healing rates of bone [15] as well as by pain symptoms. Approximately six weeks' post intervention pain ceased when walking slowly. Three months postoperative she reported pain free sitting and walking distance of 500 m. Subsequent rehabilitation focused on functional movement, flexibility and strength training of abdominals, hips and lower extremity. Pain presentation and clinical findings 6 months postoperative was normal ROM and slight palpation tenderness on the ischial tubercle. Pain free squats, lunges, and slight tenderness during kicking and fast cutting movements. 17 months after the trauma, and 8 months postoperative she was cleared for a slow return to football, being pain free in running, cycling and sitting. Lower Extremity Functional Scale had improved to 77/80 as illustrated in Fig. 3.

## 2 Discussion

**Fig. 4:**
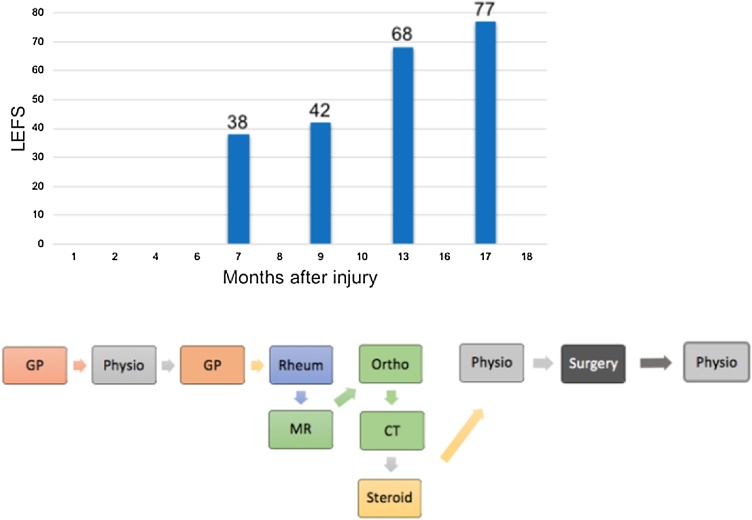
Lower Extremity Functional Scale and timeline with overview of patient’s consultations and specialist’s involvement.

This is the first paper to present percutaneous K-wire fenestration of the non-union fracture of the ischial tuberosity in an adolescent girl. The findings of this case report suggest that conservative treatment was well warranted in the initial stages as there was a functional improvement. A second period of conservative treatment warranted no further improvement, where after surgical procedure approximately nine months after the initial injury made full recovery possible (Fig. 4).

Singer and colleagues [16] assumed avulsion fractures with a displacement >15 mm can be considered an indication for open reduction and osteosynthesis, whereas patients with a fragment displacement <15 mm can be treated conservatively and an excellent outcome can be expected. The novelty of the percutaneous K-wire method, in repairing a non-union may therefore be seen as a possible supplement to avulsion fractures with a displacement <15 mm, where conservative treatment has shown to be unsuccessful. The displacement fracture margins however do differ in trials, as Shuett et al. [17] mention displacement margins of 20 mm. There may therefore be a need for consensus of displacement fractures margins, for the sake of being able to compare studies and interventional methods.

Eberlach and colleagues [18] found the overall success and return to sports rate was higher in the patients receiving surgery (88%), compared to conservative treatment (79%) and patients with fragment displacement greater 15 mm and high functional demands, surgical treatment should be considered.

We have demonstrated that this novel surgical method may be preferred to displacements less than 15 mm when conservative treatment is ineffective. This does however warrant further investigation. We are not able to establish if earlier surgery could have reduced time to rehabilitation and return to sport. However when a reasonable conservative treatment period of 2–4 months (with an initial period of rest is ineffective and the patient has a delayed union, surgery could be considered [5]. This decision may have been possible if imaging had been initiated earlier in the process, thereby confirming the diagnosis at an earlier stage.

1 Avulsion fracture is always a differential diagnosis in proximal hamstring pain in adolescence.
2 In most acute cases standard radiographs of the pelvis are sufficient, however in nondistinctive cases, CT, MRI, or even ultrasound may help to make the correct diagnosis.
3 Consider fenestration of avulsion fractures if no or small progression with conservative treatment after 3–4 months after injury.

Informed consent for publishing the study was given by the parents, as the patient is a minor.

We further confirm that no ethical approval was needed for completing this retrospective case report. (The National Committee on Health Research Ethics. http://www.nvk.dk/English). The case report is anonymous and has no intention of research, but describes a treatment offered at the University Hospital xxxx Denmark. The parent of the patient involved (a minor) has in writing accepted the publication of the case study.

## Conflicts of interest

We wish to confirm that there are no known conflicts of interest associated with this publication and there has been no significant financial support for this work that could have influenced its outcome.

## Funding

None.

## Ethical approval

We further confirm that no ethical approval was needed for completing this retrospective case report. (The National Committee on Health Research Ethics. http://www.nvk.dk/English). The case report is anonymous and has no intention of research, but describes a treatment offered at the University Hospital Aalborg, Aalborg, Denmark. The parent of the patient involved (a minor) has in writing accepted the publication of the case study.

## Consent

Written consent to publish the anonymous case report of a minor, was obtained from the mother to the child.

## Author contribution

Jens Erik Jorgensen (JEJ) carried out the early rehabilitation before and after surgery.

Jens Kristinsson (JK) carried out the surgical procedure.

Carsten M. Mølgaard (CMM) did the initial examinations and follow-up at the orthopaedic sports clinic.

All authors participated in drafting the manuscript and approved the final manuscript.

## Registration of research studies

UIN: Research registry 4404.

## Guarantor

Jens Erik Jorgensen, jeja26@gmail.com.

Carsten M. Mølgaard, cmm@rn.dk.

Jens Kristinsson, hjk@rn.dk.

## Provenance and peer review

Not commissioned, externally peer reviewed.
